# Stone ablation efficacy: a comparison of a thulium fibre laser and two pulse-modulated holmium:YAG lasers

**DOI:** 10.1007/s00240-022-01393-4

**Published:** 2023-01-12

**Authors:** Bingyuan Yang, Aditi Ray, Jian James Zhang, Steven Peng, Mike O’Brien, Ben Turney

**Affiliations:** 1 Nuffield Department of Surgical Sciences, University of Oxford, Oxford, UK; 2 Boston Scientific, Marlborough, MA USA

**Keywords:** Lithotripsy, Ablation rate, Endourology, Laser

## Abstract

We present preliminary stone ablation rate results from an automated bench model using two pulse-modulated Ho:YAG lasers and a thulium fibre laser (TFL) in contact and non-contact modes. Ablation rate was assessed using automated apparatus that moved the laser fibre across flat BegoStone phantoms at a constant stone-to-fibre working distance (WD). Pre-soaked and unsoaked stones were used. A range of powers (20–60 W) was tested at WD of up to 3 mm. In pseudocontact, the prototype Ho:YAG laser produced higher ablation than the reference Ho:YAG laser at all powers tested (*p* < 0.002), and higher ablation than TFL at 20 W and 40 W (*p* < 0.001). At distance, ablation rates for the prototype were higher than the reference Ho:YAG laser using pre-soaked stones at WD up to 3 mm (*p* < 0.001). TFL required the laser fibre to be moved faster (5–12 mm/s) for optimal ablation, compared to 1–3 mm/s for the Ho:YAG lasers. TFL was unable to demonstrate ablation with unsoaked BegoStone. At any given power, similar ablation rates were achievable with all three lasers under optimised conditions. Novel pulse-modulation modes demonstrated higher ablation rates than the reference Ho:YAG laser’s pulse-modulation at a range of powers and WDs. Ablation rate of Ho:YAG lasers decreased linearly with WD whereas the ablation rate of TFL decreased rapidly beyond 2 mm WD. TFL was more affected by scan speed and pre-soaking of stone than Ho:YAG lasers. Ho:YAG lasers may be more practical in clinical settings because they are less dependent on ablation technique.

## Introduction

Medical lasers have revolutionised intracorporeal lithotripsy. Today’s key players are the Ho:YAG laser and the more recent thulium fibre laser (TFL), and pulse-modulation technologies can further alter how each of these lasers interact with stones as well as the intervening liquid medium.

One of the key parameters for laser efficacy is the rate of stone ablation. A higher ablation rate results in shorter procedures, which in turn is associated with fewer complications [2]. In ideal conditions the laser fibre should be in contact with the stone to minimise attenuation of laser energy, but in practice this usually cannot be maintained continuously and half or more of laser pulses may be delivered when the fibre is more than 0.5 mm from the stone [3]. Therefore, ablation efficacy is important whether the fibre is in contact with the stone or not.

In vitro data has been reported on the ablation rate of various Ho:YAG and TFL lasers using specific types of human urinary calculi [4, 5]. However, a trade-off of using these authentic stones is that their irregular shape requires a human operator and precludes automated, operator-independent bench testing. We have, therefore, used BegoStone phantoms which have been shown to have comparable physical properties to urinary calculi [6].

We present stone ablation rate results from an in-vitro, automated bench model, using two different Ho:YAG lasers and a TFL in both contact and non-contact modes. The settings were optimised for each laser. This provides insight into how different laser types and settings perform under different test conditions using a consistent methodology.

## Methods

### Lasers evaluated

Three lasers have been selected for the initial experiments: a reference Ho:YAG pulse modulated laser, a reference TFL and a prototype holmium:YAG pulse modulated laser.

The reference Ho:YAG laser (“RHL”) provides standard functionality in the form of short, medium and long pulse firing modes, as well as two pulse-modulated firing modes designed for contact firing (contact, C) and non-contact firing (distance, D). A compatible 230 μm laser fibre was used.

The reference TFL (“TFL”) is a 60 W laser with a high peak power of 500 W. It provides standard functionality in the form of short, medium and long pulse firing modes. A compatible 200 μm laser fibre was used.

The prototype Ho:YAG laser (“prototype”) has been selected to represent a new form of pulse modulation. It provides standard functionality in the form of short and long pulse firing modes, as well as two novel pulse-modulated firing modes (pulse-modulated 1, PM1 and pulse-modulated 2, PM2). A compatible 242 μm prototype laser fibre was used.

### Ablation rate

Ablation rate was assessed using two methods: pseudocontact and distance. The “pseudocontact” experiment measures the ablation rate when the laser fibre is as close to the stone phantom as possible, and the “distance” experiment measures the effect of increased fibre-to-stone working distance (WD) on ablation rate. BegoStone phantoms were used to provide a flat stone surface and maintain a consistent fibre-to-stone WD. These used a 15:3 ratio of BegoStone Plus powder to deionised water. They were then dried at room temperature for 24 h, and the initial dry weight measured. One set of stones was soaked in water for 24 h prior to ablation (“pre-soaked”) and one set was left dry until the time of ablation (“unsoaked”).

**Fig. 1:**
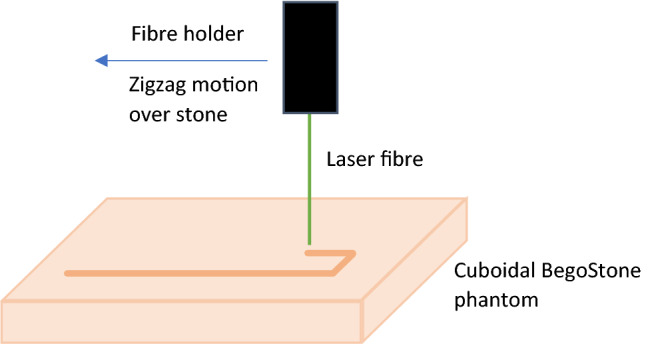
Ablation rate experiment overview. The laser fibre is scanned across the surface of the BegoStone phantom in a zig-zag pattern at a constant speed and WD

All ablation rate experiments used automated apparatus that held the laser fibre perpendicular to the surface of a flat BegoStone phantom as shown in Fig. 1. The fibre was scanned across the surface of the stone by a motorised arm, which maintained a constant speed of motion and WD. The total fibre movement was 110 mm in all cases, while the speed of motion was adjusted for different combinations of lasers and settings to maximise the ablation rate. This mimics the way that an operator might slow down in response to a lower power setting or a particularly hard stone. The stone and fibre tip were immersed in 0.9% sodium chloride throughout and there was continuous irrigation to wash away dust and fragments.

The ablated stone phantoms were dried at room temperature for 24 h and re-weighed. The ablation rate was then calculated as follows.

$$Ablation rate= \frac{\Delta\, dry\, mass}{time}$$

The laser fibre was stripped and cleaved after each experiment and the power output was re-checked using a power meter (Ophir Optronics).

### Ablation in pseudocontact

Preliminary experiments showed that the optimal WD for pseudocontact experiments is 0.5 mm. Closer proximity puts the fibre at risk of coming into contact with small imperfections on the stone surface, disrupting the ablation as well as exacerbating fibre damage known as “burnback” [7].

Laser settings in pseudocontact were selected at 20, 40 and 60 W as shown in Table 1. These represent settings that could be used clinically at those power levels for dusting and fragmentation, and which gave the highest ablation at those power levels in preliminary testing.

**Table 1:** Laser settings for pseudocontact experiments. Approximate energies of each micropulse are given in parentheses

Laser	20 W		40 W		60 W	
RHL dust (C)	0.4 J 50 Hz		1 J 40 Hz		2 J 30 Hz	
RHL frag (D)	1 J 20 Hz		1 J 40 Hz		2 J 30 Hz	
TFL dust (short pulse)	0.2 J 100 Hz		0.2 J 200 Hz		0.2 J 300 Hz	
TFL frag (short pulse)	0.5 J 40 Hz		0.5 J 80 Hz		0.5 J 120 Hz	
Prototype dust (PM1)	1 J 20 Hz	(42 mJ)	2 J 20 Hz	(83 mJ)	3 J 20 Hz	(94 mJ)
Prototype frag (PM2)	1 J 20 Hz	(143 mJ)	2 J 20 Hz	(286 mJ)	3 J 20 Hz	(429 mJ)

Both the RHL and prototype lasers used pulse-modulated firing modes in these experiments. For the prototype laser, the pulse energy represents the total energy across all micropulses within each pulse. The energy of each individual micropulse is given in parentheses.

**Fig. 2:**
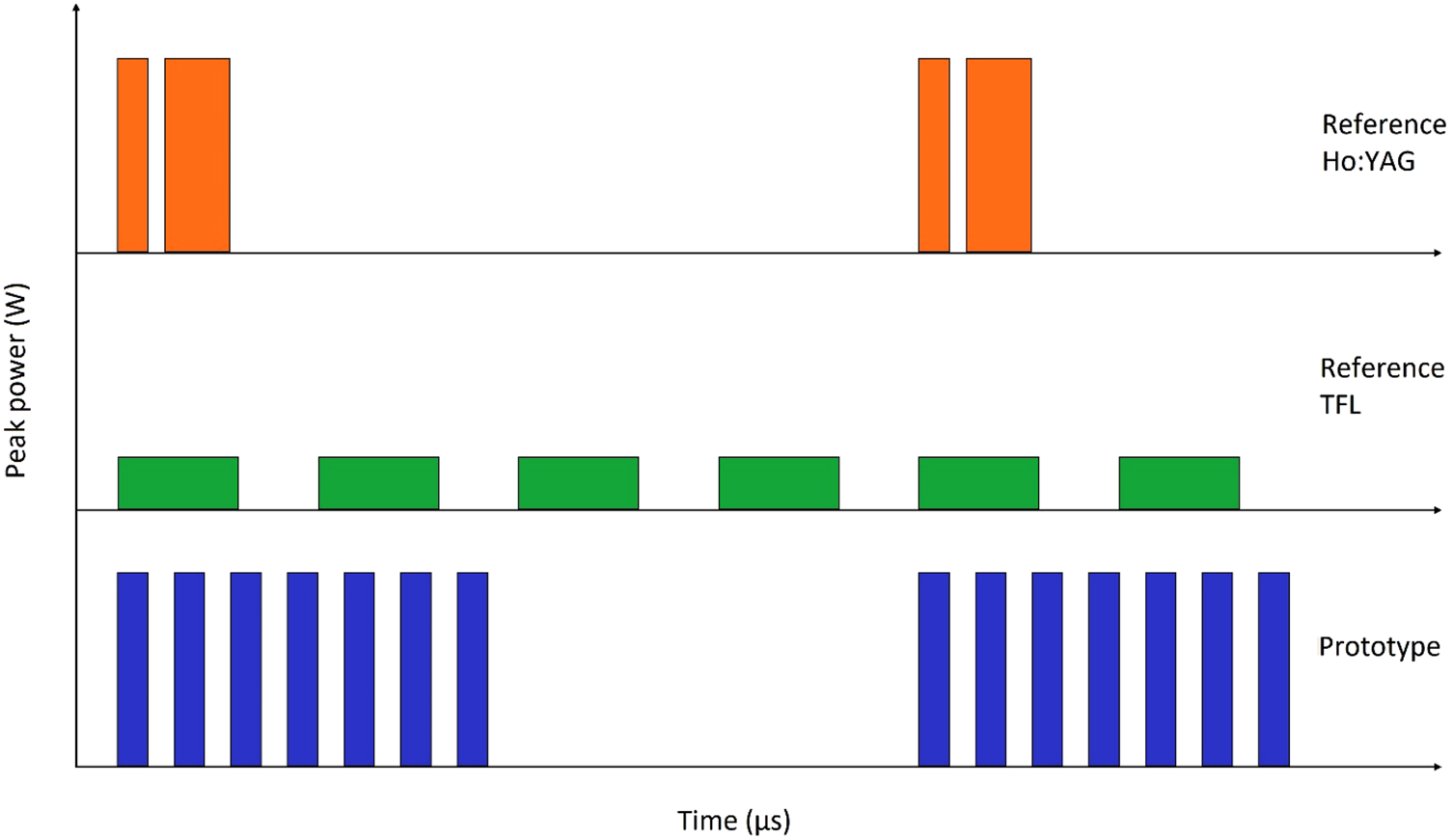
Laser pulse profile examples. The reference Ho:YAG laser pulse-modulation splits each pulse into two closely spaced power peaks. The TFL produces longer pulses with lower peak powers. The prototype Ho:YAG laser pulse-modulation splits each pulse into 7–36 equal micropulses. Not to scale

RHL pulse-modulated firing modes split each pulse into 2, while the prototype laser pulse-modulated firings modes have 24–36 micropulses per pulse in PM1 mode (optimised for dusting) and 7 micropulses per pulse in PM2 mode (optimised for fragmentation). For example, at 3 J 20 Hz in PM1 mode, the prototype laser delivers 32 micropulses of 94 mJ in each pulse, at a rate of 20 pulses per second. The net result is that 3 J 20 Hz PM1 mode delivers 640 micropulses per second of 94 mJ each. Figure 2 illustrates the different pulse profiles of each laser for the fragmentation settings used.

### Scan speed

The scan speed is the speed at which the laser fibre was moved across the surface of the stone phantom. The optimal scan speed for each laser system and setting was determined in preliminary experiments by starting at 1 mm/s and increasing the scan speed until an increase in scan speed no longer resulted in an increase in ablation rate. These optimal scan speeds are shown in Table 2.

**Table 2:** Scan speeds for pseudocontact experiments

Laser	20 W		40 W		60 W	
RHL dust (C)	0.4 J 50 Hz	1 mm/s	1 J 40 Hz	1 mm/s	2 J 30 Hz	2 mm/s
RHL frag (D)	1 J 20 Hz	1 mm/s	1 J 40 Hz	1 mm/s	2 J 30 Hz	2 mm/s
TFL dust (short pulse)	0.2 J 100 Hz	1 mm/s	0.2 J 200 Hz	5 mm/s	0.2 J 300 Hz	12 mm/s
TFL frag (short pulse)	0.5 J 40 Hz	1 mm/s	0.5 J 80 Hz	2 mm/s	0.5 J 120 Hz	8 mm/s
Prototype dust (PM1)	1 J 20 Hz	1.5 mm/s	2 J 20 Hz	1.5 mm/s	3 J 20 Hz	1.5 mm/s
Prototype frag (PM2)	1 J 20 Hz	1 mm/s	2 J 20 Hz	2.5 mm/s	3 J 20 Hz	3 mm/s

### Ablation at distance

Ablation at a distance was measured by performing the ablation rate experiment at WDs of 0.5 mm, 1 mm, 2 mm, 3 mm and 4 mm. If no visible ablation was seen then the WD was reduced by 0.5 mm. Only results with visible ablation are included.

Laser settings at a distance were selected at 40 W as shown in Table 3. Exact 40 W settings were not available for the prototype laser experiments with unsoaked stones, so 35 W and 45 W settings were used instead.

**Table 3:** Laser settings for distance experiments. Approximate energies of each micropulse are given in parentheses

Laser	Laser settings at a distance
RHL (D)	1 J 40 Hz = 40 W
TFL (short pulse)	0.2 J 200 Hz = 40 W
Prototype (PM2)	2 J 20 Hz = 40 W (286 mJ)
Prototype, unsoaked (PM2)	3.5 J 10 Hz = 35 W (500 mJ) and 3 J 15 Hz = 45 W (429 mJ)

### Statistical methods

This is a hypothesis-generating study and analyses were exploratory in nature. 9 repetitions were performed for each combination of laser settings, and the arithmetic mean, standard deviation and 95% confidence interval were calculated. Post-hoc statistical analysis was performed by pairwise comparison of ablation rates using a two-sample two-tailed t-test, with no adjustment for multiple comparisons.

## Results

### Ablation in pseudocontact

**Fig. 3:**
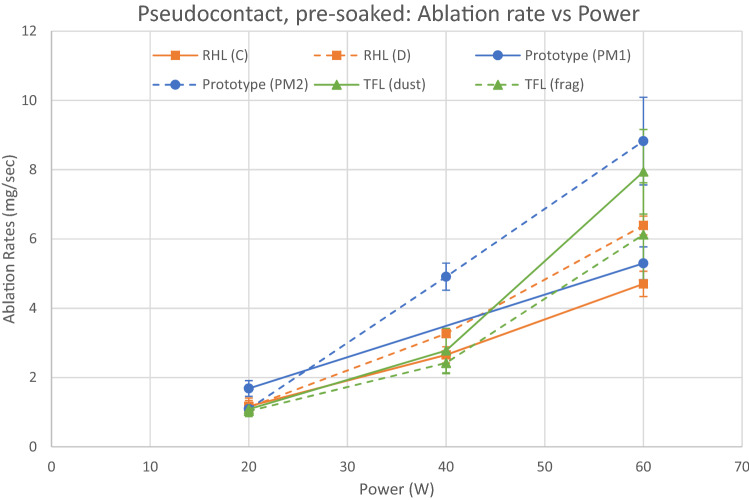
Ablation in pseudocontact, pre-soaked stones. Error bars show the 95% CI. See disclaimers*

Pseudocontact ablation rates for pre-soaked stones are shown in Fig. 3 and Table 4.

**Table 4:** Ablation rate in pseudocontact, pre-soaked stones. Statistically significant differences (*p* < 0.05) are in bold, calculated by two-sided t-test. See disclaimers*

Laser and power 20 W	Ablation rate				Vs laser (*p* values)					
	Mean (mg/s)		S.D. (mg/s)	95% CI (mg/s)	RHL dust	RHL frag	TFL dust	TFL frag	Proto. dust	Proto. frag
RHL dust (C)	1.170		0.309	0.933–1.407	–	0.837	0.465	0.282	**0.002**	0.496
RHL frag (D)	1.142		0.255	0.946–1.338	0.837	–	0.564	0.328	**< 0.001**	0.606
TFL dust (short pulse)	1.089		0.085	1.023–1.154	0.465	0.564	–	0.459	**< 0.001**	0.921
TFL frag (short pulse)	1.032		0.206	0.874–1.190	0.282	0.328	0.459	–	**< 0.001**	0.440
Prototype dust (PM1)	1.684		0.295	1.457–1.910	**0.002**	**< 0.001**	**< 0.001**	**< 0.001**	–	**< 0.001**
Prototype frag (PM2)	1.093		0.105	1.012–1.174	0.496	0.606	0.921	0.440	**< 0.001**	–
40 W										
RHL dust (C)	2.655		0.303	2.423–2.888	–	** < 0.001**	0.685	0.178	–	**< 0.001**
RHL frag (D)	3.268		0.117	3.177–3.358	**< 0.001**	–	0.118	**< 0.001**	–	**< 0.001**
TFL dust (short pulse)	2.778		0.831	2.139–3.417	0.685	0.178	–	0.268	–	**< 0.001**
TFL frag (short pulse)	2.420		0.398	2.114–2.726	0.178	**< 0.001**	0.268	–	–	**< 0.001**
Prototype dust (PM1)	–		–		–	–	–	–	–	–
Prototype frag (PM2)	4.912		0.509	4.520–5.303	**< 0.001**	**< 0.001**	**< 0.001**	**< 0.001**	–	–
60 W										
RHL dust (MC)	4.703		0.473	4.340–5.067	–	**< 0.001**	**< 0.001**	0.062	**0.036**	**< 0.001**
RHL frag (MD)	6.390		0.350	6.121–6.659	**< 0.001**	–	**0.019**	0.699	**< 0.001**	**0.002**
TFL dust (short pulse)	7.941		1.588	6.720–9.161	**< 0.001**	**0.019**	–	**0.047**	**0.001**	0.241
TFL frag (short pulse)	6.127		1.945	4.632–7.622	0.062	0.699	**0.047**	–	0.251	**0.005**
Prototype dust (PM1)	5.298		0.616	4.824–5.772	**0.036**	**< 0.001**	**0.001**	0.251	–	**< 0.001**
Prototype frag (PM2)	8.826		1.645	7.604–10.132	**< 0.001**	**0.002**	0.241	**0.005**	**< 0.001**	–

Statistically significant differences (*p* < 0.05) are in bold

All lasers demonstrated a linear relationship between total power and ablation rate.

At 20 W, the prototype laser in PM1 mode produced a higher ablation rate (*p* < 0.002) than all other settings, which were not significantly different.

At 40 W, the prototype laser in PM2 mode produced a higher ablation rate (*p* < 0.001) than all other settings, which were not significantly different.

At 60 W, the prototype laser in PM2 mode and TFL with dusting settings produced higher ablation rates (prototype: *p* < 0.005; TFL: *p* < 0.047) than all other settings, which were not significantly different.

### Ablation at distance

**Fig. 4:**
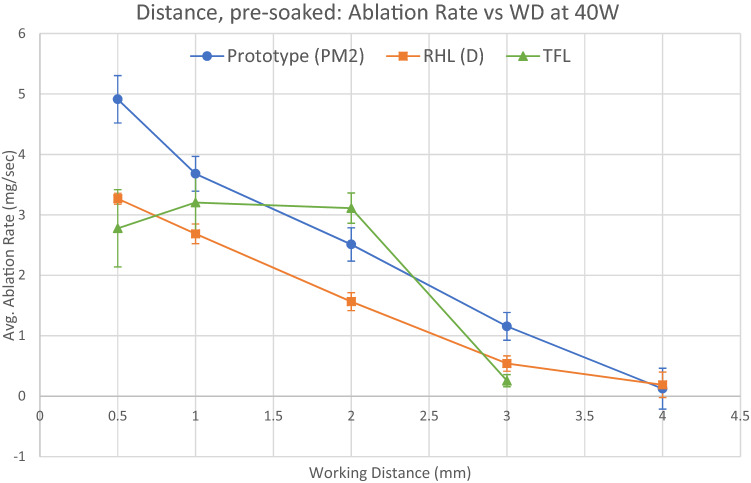
Ablation at distance, pre-soaked stones. Error bars show the 95% CI. See disclaimers*

Ablation at distance results for pre-soaked stones using 40 W settings are shown in Fig. 4 and Table 5. Both Ho:YAG lasers were using their respective pulse-modulated firing modes optimised for ablation at distance. The TFL was operating in short pulse mode, which we found to be optimal for this purpose.

**Table 5:** Ablation rate at distance, pre-soaked stones. Statistically significant differences (*p* < 0.05) are in bold, calculated by two-tailed t test. See disclaimers*

Laser and WD	Ablation rate			Vs laser (*p* values)		
	Mean (mg/s)	S.D. (mg/s)	95% CI (mg/s)	RHL	TFL	Prototype
0.5 mm						
RHL (D)	3.268	0.117	3.177–3.358	–	0.118	**< 0.001**
TFL (short pulse)	2.778	0.831	2.139–3.417	0.118	–	**< 0.001**
Prototype (PM2)	4.912	0.509	4.520–5.303	**< 0.001**	**< 0.001**	–
1 mm						
RHL (D)	2.686	0.211	2.524–2.849	–	**0.039**	**< 0.001**
TFL (short pulse)	3.205	0.619	2.728–3.681	**0.039**	–	0.070
Prototype (PM2)	3.680	0.373	3.393–3.967	**< 0.001**	0.070	–
2 mm						
RHL (D)	1.565	0.193	1.416–1.713	–	**< 0.001**	**< 0.001**
TFL (short pulse)	3.111	0.326	2.861–3.362	**< 0.001**	–	**0.002**
Prototype (PM2)	2.511	0.358	2.235–2.786	**< 0.001**	**0.002**	–
3 mm						
RHL (D)	0.541	0.165	0.414–0.668	–	**0.001**	**< 0.001**
TFL (short pulse)	0.257	0.130	0.157–0.357	**0.001**	–	**< 0.001**
Prototype (PM2)	1.155	0.299	0.925–1.385	**< 0.001**	**< 0.001**	–
4 mm						
RHL (D)	0.189	0.286	-0.149–0.527	–	–	0.129
TFL (short pulse)	–	–		–	–	–
Prototype (PM2)	0.124	0.199	-0.028–0.277	0.129	–	–

Statistically significant differences (*p* < 0.05) are in bold

All lasers were able to ablate effectively at WDs of up to 2 mm. Ablation rate for the two Ho:YAG lasers decayed approximately linearly with WD but was measurable at 3 mm WD. Ablation rate for TFL remained approximately static up to 2 mm WD but rapidly fell off after that.

At all WDs up to 3 mm, the prototype laser produced a higher ablation rate than RHL (*p* < 0.001).

TFL produced a higher ablation rate than both Ho:YAG lasers at 2 mm WD (prototype: *p* = 0.002; RHL: *p* < 0.001) but a lower ablation rate at 3 mm WD (prototype: *p* < 0.001; RHL: *p* = 0.001).

**Fig. 5:**
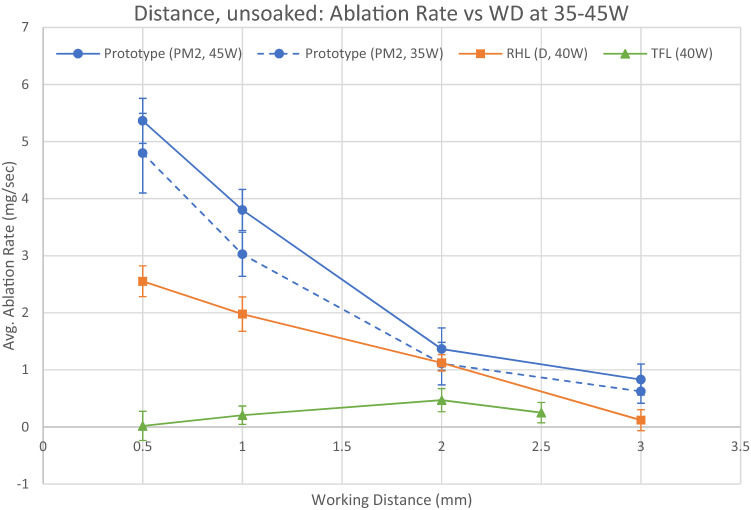
Ablation at distance, unsoaked stones. Error bars show the 95% CI. See disclaimers*

Ablation at distance results for unsoaked stones using 35-45 W settings are shown in Fig. 5 and Table 6.

**Table 6:** Ablation rate at distance, unsoaked stones. Statistically significant differences (*p* < 0.05) are in bold, calculated by two-tailed t test. See disclaimers*

Laser and WD	Ablation rate			Vs laser (*p* values)			
	Mean (mg/s)	S.D. (mg/s)	95% CI (mg/s)	RHL	TFL	Prototype 35 W	Prototype 45 W
0.5 mm							
RHL (D)	2.553	0.351	2.283–2.822	–	**< 0.001**	**< 0.001**	**< 0.001**
TFL (short pulse)	0.018	0.333	− 0.237 to 0.274	**< 0.001**	–	**< 0.001**	**< 0.001**
Prototype (35 W, PM2)	4.797	0.907	4.107–5.501	**< 0.001**	**< 0.001**	–	0.116
Prototype (45 W, PM2)	5.363	0.512	4.994–5.784	**< 0.001**	**< 0.001**	0.116	–
1 mm							
RHL (D)	1.988	0.392	1.677–2.279	–	**< 0.001**	** < 0.001**	**< 0.001**
TFL (short pulse)	0.206	0.210	0.044–0.367	**< 0.001**	–	** < 0.001**	**< 0.001**
Prototype (35 W, PM2)	3.026	0.504	2.650–3.423	**< 0.001**	**< 0.001**	–	**0.003**
Prototype (45 W, PM2)	3.802	0.464	3.463–4.183	**< 0.001**	**< 0.001**	**0.003**	–
2 mm							
RHL (D)	1.124	0.186	0.981–1.267	–	**< 0.001**	0.952	0.181
TFL (short pulse)	0.470	0.264	0.267–0.672	**< 0.001**	–	**0.004**	**< 0.001**
Prototype (35 W, PM2)	1.110	0.485	0.741–1.485	0.952	**0.004**	–	0.274
Prototype (45 W, PM2)	1.365	0.479	1.001–1.741	0.181	**< 0.001**	0.274	–
3 mm							
RHL (D)	0.119	0.239	−0.065 to 0.303	–	–	**< 0.001**	**< 0.001**
TFL (short pulse)	–	–		–	–	–	–
Prototype (35 W, PM2)	0.623	0.272	0.417–0.831	**< 0.001**	–	–	0.178
Prototype (45 W, PM2)	0.830	0.354	0.562–1.105	**< 0.001**	–	0.178	–

Statistically significant differences (*p* < 0.05) are in bold

TFL was unable to effectively ablate the unsoaked BegoStone phantom in this experiment. Instead of ablating a trench, the stone surface was discoloured to a pale tan colour without any apparent removal of stone or dust production, and no visible effect at all at beyond 2.5 mm WD. The calculated ablation rate for TFL with unsoaked stones was 0.06–15% of the equivalent ablation rate using pre-soaked stones.

The two Ho:YAG lasers produce a lower ablation rate when unsoaked stones are used, with the RHL producing an ablation rate of 22–78% of the equivalent ablation rate using pre-soaked stones. The difference is more pronounced at longer WDs. In contrast to TFL, the trenches looked visually similar with pre-soaked and unsoaked stones when Ho:YAG lasers were used.

### Impact of scan speed

TFL required higher scan speeds for an optimal ablation rate than either Ho:YAG laser. In pseudocontact, optimal TFL scan speeds were up to 12 mm/s, compared to 1-3 mm/s for the Ho:YAG lasers. For example, using 0.5 J 120 Hz (60 W) settings at a scan speed of 2 mm/s resulted in an ablation rate of 1.97 mg/s compared to 6.13 mg/s at the optimal scan speed of 8 mm/s.

At higher powers of 50-60 W, the RHL benefitted from scan speeds of up to 2 mm/s but both Ho:YAG lasers saw a reduced ablation rate at scan speeds above 3 mm/s. Conversely, the TFL saw continued gains in ablation rate at scan speeds of 8–12 mm/s depending on the setting used.

Results with ablation at a distance were similar, with TFL requiring higher scan speeds of up to 5 mm/s compared to up to 2.5 mm/s (prototype) and 1 mm/s (RHL).

## Discussion

These results demonstrate that when experimental conditions such as scan speed, WD and stone hydration are optimised for each laser, all three lasers are capable of producing broadly similar ablation efficacy on BegoStone phantoms across a range of clinical power settings. Between the Ho:YAG lasers. the prototype laser produced a higher ablation rate than the RHL in a number of settings. As both of these lasers operate at the same 2120 nm wavelength, these differences should reflect advantages of the novel pulse modulation technology used in the prototype over the reference Ho:YAG laser’s pulse modulation. There may be scope for this technology to produce further increases in the ablation rate without requiring additional energy delivery.

The TFL required greater experimental optimisation to achieve the best results. The most striking difference in requirements was the inability of the TFL to ablate unsoaked BegoStone. This has been reported previously [9]. Both Ho:YAG lasers also performed less well with unsoaked stone but to a much lesser extent. It is unclear how relevant this difference is in clinical use, where in theory all stones will be hydrated in the urine. However, there are anecdotal reports of TFLs charring or melting stones in clinical use which may be a manifestation of this phenomenon. This implies a difference in the mechanism of stone ablation between TFL and Ho:YAG lasers.

It is well established that Ho:YAG lasers ablate stone through a primarily photothermal mechanism with a minimal photoacoustic (shockwave) effect [10–12]. The mechanism for TFL is not as well understood although the underlying physics would imply that the longer pulses of TFL are only capable of photothermal interactions [12]. Our findings suggest that the mechanism for TFL is more dependent on the water content within the stone matrix than that of Ho:YAG.

The TFL required much higher scan speeds to produce optimal ablation. This corresponds with other work showing that the optimal scan speed can vary between Ho:YAG and TFL as well as depending on the exact settings used [13]. These very high scan speeds equate to very rapid movement of the laser fibre by the Urologist and may be difficult or even impossible to achieve in clinical settings. In contrast, the optimal scan speed for RHL was between 1 and 2 mm/s for all settings tested, which is more achievable clinically.

At distance, the ablation rate for Ho:YAG lasers appears to decay approximately linearly when one might expect exponential decay with distance. One consideration is that fibre burnback is higher at shorter WDs, resulting in artificially lower ablation rates when the fibre is closer to the stone. This is difficult to quantify in measurements, but certainly the loss of fibre length is more pronounced when the WD is ≤ 1 mm. This may also explain why the TFL ablation rate appears to be lower in pseudocontact (with much higher variance) than at 1 mm or 2 mm WDs.

Although TFLs are generally considered contact-only lasers [14] due to their higher water absorption, we were able to demonstrate effective ablation at a 2 mm WD using a typical 0.2 J and 200 Hz setting. The water absorption coefficient of TFLs is approximately 14 mm^−1^ [15], meaning that 95% of laser energy is absorbed by about 0.2 mm of water. This implies that the TFL ablation rates at the distance demonstrated here must be the result of a Moses effect [16] where part of the laser pulse creates a water vapour bubble and reduces the attenuation of the remainder of the pulse. The TFL pulse is known to be much longer than equivalent Ho:YAG pulses [17] and it’s possible that the resulting stream of connecting bubbles [18] produces a more pronounced Moses effect than previously thought.

This model allows bench comparison of different lasers under optimised conditions while controlling key variables such as stone consistency, scan speed and WD. Further experiments should be conducted in anatomical models of kidneys or ureters, using human stone as well as suitable stone phantoms, to investigate the extent to which these optimal settings can be applied in these scenarios where motion may be restricted by anatomy or the manoeuvrability of a ureteroscope.

## Conclusion

At any given power, similar ablation rates were achievable with the three lasers evaluated in this study, provided technique and stone factors were optimised. The novel pulse modulation modes of the prototype laser demonstrated higher ablation rates than the reference Ho:YAG laser’s pulse modulation at a range of powers and WDs. Next-generation pulse modulation technology may allow further improvements in ablation rate without requiring additional power.

TFL required higher scan speeds for optimal ablation rate (up to 12 mm/s), and was more affected by scan speed and pre-soaking of stone than Ho:YAG lasers. This may reflect a difference in the underlying mechanism of ablation between Ho:YAG and TFL.

The ablation rate of the Ho:YAG lasers decreased approximately linearly with working distance whereas the ablation rate of the TFL decreased rapidly beyond a 2 mm WD.

These in vitro ablation studies suggest that the Ho:YAG lasers may have better ablation efficiency in a clinical setting because they are less dependent on ablation technique.

## Disclaimers

*Bench test results may not necessarily be indicative of clinical performance. The prototype laser is a concept device or technology and is not yet available for sale.

## Data Availability

The datasets generated during and/or analysed during the current study are available from the corresponding author on reasonable request.
